# Novel Study on Cryogenic Distillation Process and Application by Using CHEMCAD Simulation

**DOI:** 10.1021/acsomega.3c09490

**Published:** 2024-03-25

**Authors:** Hüseyin Berat Erdöl, Fatma İrem Şahin, Nil Acaralı

**Affiliations:** Department of Chemical Engineering, Yildiz Technical University, Esenler-Istanbul 34220, Türkiye

## Abstract

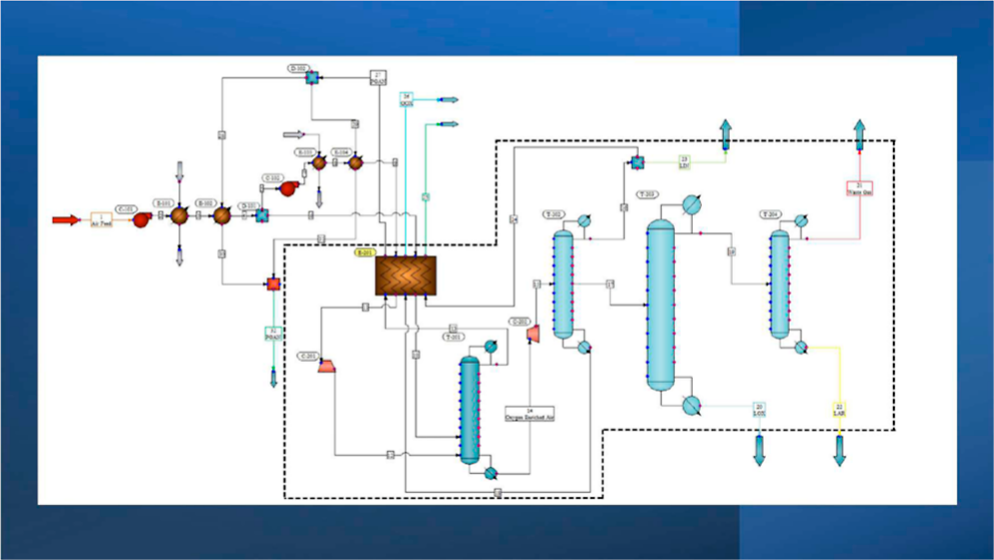

The purpose of this study was to examine the cryogenic separation process used in air separation plants with the CHEMCAD simulation program. A preliminary analysis was carried out with the literature about the operation of the process. In light of the technical information, the simulation of the cryogenic separation process was utilized with the program. While no changes were analyzed in the basic parts of the process, the filtration of the air and separation from moisture and carbon dioxide were not included in the simulation. The simulation showed the three basic components of air, nitrogen, oxygen, and argon obtained as a product of the desired purity rather than a one-to-one demonstration of the applications in the industry. In addition, the low-temperature separation process of an air separation unit was studied to obtain high-purity products (99.49–99.99%) achieving the expected separation efficiency. As a result, the production of all three substances in desired purity was achieved successfully.

## 1 Introduction

Energy is a crucial component in both the pumping and treatment stages of the water cycle. Pumping energy is contingent on factors such as distance, flow rate, and friction, while the energy demands of the desalination process hinge on source water quality, contamination characteristics, and chosen processes. Significantly, fossil fuel-driven, energy-intensive desalination processes represent a principal source of CO_2_ emissions. Currently, the installed global desalination capacities contribute 76 million tons of CO_2_ annually, with expectations of reaching 218 million tons per year by 2040.^1^

In a study, there had extensive development and analysis of carbon capture and storage techniques emerging as a viable alternative to mitigate the release of CO_2_ into the atmosphere.^2^ In the literature, the pilot-scale unit for CO_2_ separation and compression served as an outstanding test platform for investigating the influence of flue gas impurities on the process of CO_2_.^3^ In the process, a revolutionary development enabled the production not only of oxygen gas but also of nitrogen gas which constituted over three-quarters of the atmosphere.^4^ Later, it became possible to obtain argon gas which was the third largest in air in terms of volume but considerably lower than the other two gases.^5^ The process called cryogenic distillation also known as cryogenic rectification or liquefaction of air had a unique design that allowed all three gases to be obtained with comparison to other air separation processes mentioned in the literature. It made it possible to separate nitrogen and oxygen by liquefying at very low temperatures with the integrated double distillation column system, one high-pressure column and the other low-pressure column inside. The features of this system which could be examined in more detail in the continuation of the study in terms of efficiency.^6^ In industrial scope, it had the largest share in the pie in terms of air separation process applications.^7^ To understand the value of the products obtained after the separation process, it would be better to start the study from the properties of nitrogen, oxygen, and argon gases and their uses in industry.^8^ Several alternative methods for the air separation process were argued in the study.^9^ Each of the different methods distinguished by working principles, equipment sets, and production efficiencies.^10^ However, each type of process shared the main purpose of separating air into its three most common components: N_2_, O_2_, and Ar.^11^ As it could be explained in further reading, some of the processes lacked the ability of separating each component from air but successfully separated the desired compound.^12^ Thus, selection between the methods required knowledge of the process outputs. The cryogenic distillation method is a unique process among the others in terms of producing each of the three compounds in a single process setup.^13^ In a study, it was aimed to demonstrate and compare alternative oxygen production methods with a comparing method of multicriteria decision-making methods. With this technique, it was possible to rank oxygen production methods in terms of performance, cost, and safety. According to this study, oxygen production with membrane technology gave the optimal results.^14^ In a paper, air separation techniques were investigated in detail. Authors categorized air separation techniques as cryogenics and noncryogenics. Essentials of each process were given with their process schemes. Also, possible improvements on the economics for the processes were proposed.^15^

Comparison between the cryogenic process and membrane method for oxygen production was evaluated. A production cycle based on ceramic membranes with a highly efficient energy cycle was searched to make a comparison. The cycle initially worked in cryogenic air separation.^16^

In the literature, air separation methods were informative. General properties of each process for air separation and cryogenic process were evaluated.^17^ Another paper investigated a system for oxygen production which was an alternative for the cryogenic process of oxygen production. Effects of oxygen concentration, temperature, oxygen flow, and pressure on efficiency and final performance of an integrated system were studied by the authors.^18^ Another alternative method for the cryogenic separation process which was the chemical looping process to produce nitrogen from air is studied in the paper. The results demonstrated good performance and favorable energy requirements of the fixed-bed configuration compared with existing nitrogen production technologies.^19^

In this study, materials for the process were determined, and cryogenic separation was explained. Then, a simulation of cryogenic process of air separation was simulated. Some of the equipment had to be replaced or removed due to the capabilities of the simulation program. For instance, the filtration section of the process at the beginning was removed while simulating the process. The aim of this simulation was to examine the feasibility of separating air into its components by obtaining low temperature and high purity in simulation programs such as CHEMCAD.

## 2 Cryogenic Distillation Mechanism

Cryogenic distillation is a separation process that exploits the differences in boiling points of components in a mixture. In air separation, the feed air was cooled to very low temperatures, typically below the boiling points of its main components. The air was then distilled in a column where different components (nitrogen, oxygen, argon) were separated based on boiling points. The colder regions of the column condensed the gases into liquid form, allowing for the collection of pure components. The success of the cryogenic distillation process in this study indicated that the simulation based on technical knowledge and without the inclusion of additional purification steps provided the desired separation efficiency for producing high-purity nitrogen, oxygen, and argon.

### 2.1 Cooling the Feed Air

The cryogenic distillation process began with cooling of the feed air to extremely low temperatures. It was typically achieved through a series of heat exchangers and refrigeration cycles.

### 2.2 Distillation Column Operation

The cooled air was then transferred into a distillation column, which served as the key apparatus for separating its main components: nitrogen, oxygen, and argon. The distillation column was equipped with multiple trays or packing materials to facilitate the separation of components. As the air ascended the column, the temperature decreased gradually from the bottom to the top.

### 2.3 Boiling Point Differences

Nitrogen, oxygen, and argon had different boiling points at atmospheric pressure. Nitrogen had the highest boiling point, followed by oxygen and then argon. The difference in boiling points was crucial to the separation process.

### 2.4 Vaporization and Condensation

The cooled air underwent vaporization and condensation as it moved through the column. Gaseous components with lower boiling points, such as nitrogen and oxygen, vaporized at higher levels in the column where temperatures were relatively higher. Argon, having a higher boiling point, remained in liquid form until lower in the column, where temperatures were colder.

### 2.5 Fractionation of Components

The ascending vapor and descending liquid phases formed a dynamic equilibrium, allowing for the fractionation of the air components. Each component was separated based on its unique boiling point and was collected at specific points in the column.

### 2.6 Collection of Pure Components

Trays or packing materials facilitated the collection of pure nitrogen, oxygen, and argon at various stages along the height of the column. The colder regions of the column acted as condensation zones, causing the vapor to turn into liquid and enabling the collection of highly pure components.

### 2.7 Final Product Collection

The separated nitrogen, oxygen, and argon were then collected as individual products with the desired purity levels.

### 2.8 Exclusion of Filtration and Additional Steps

In the study, the focus was on the core cryogenic separation process, excluding steps such as air filtration and separation from moisture and carbon dioxide. It was concluded as a specific investigation into the cryogenic distillation mechanism’s fundamental capabilities in achieving high-purity products.

Finally, the success of the cryogenic distillation process, as simulated in the study, underscored the efficiency of this method in producing high-purity nitrogen, oxygen, and argon through careful exploitation of their differing boiling points within a well-designed distillation column.

## 3 CHEMCAD Simulation of Process

**Figure 1:**
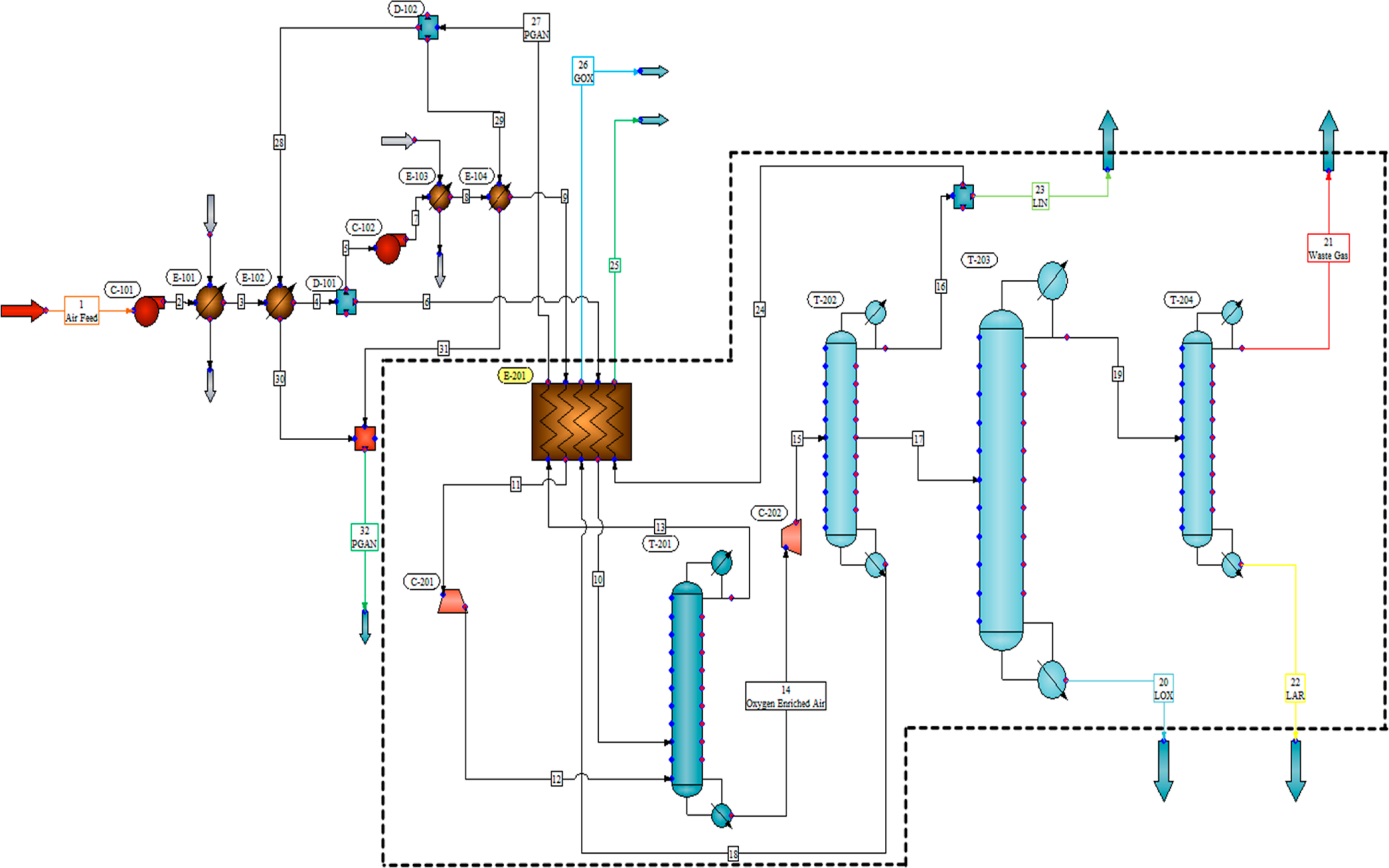
Overall process flow diagram of the air separation unit.

The overall process flowchart is given in Figure 1 (CHEMCAD version 8.0).^20^ The simulation included the introduction of the process air feed with the help of the main compressor (C-101). The temperature increased due to compression was cooled to 15–20 °C by a cooler (E-101) connected to the compressor’s output stream. Cooled air was then divided into two steams with a stream divider (D-101) with a divide ratio of 0.3. While stream 5 directly underwent the main heat exchanger (E-201), stream 4 was further compressed to 20 bar in the compressor (C-102) and then cooled to 20 °C in a cooler (E-102).

Process air was directly utilized from the atmosphere (25 °C; 1 bar). Contaminants such as dust and H_2_O and CO_2_ content were neglected. The flow rate of feed air was determined as 50,000 (StdV)m^3^/h. Overall properties of feed air is shown in Table 1.

**Table 1:** Properties of Feed

temperature (°C)	25
pressure (bar)	1
vapor fraction	1
total flow (StdVm^3^/h)	50,000
components	mole fractions
nitrogen (N_2_)	0.2095
oxygen (O_2_)	0.7810
argon (Ar)	0.0094

## 4 Results and Discussion

**Figure 2:**
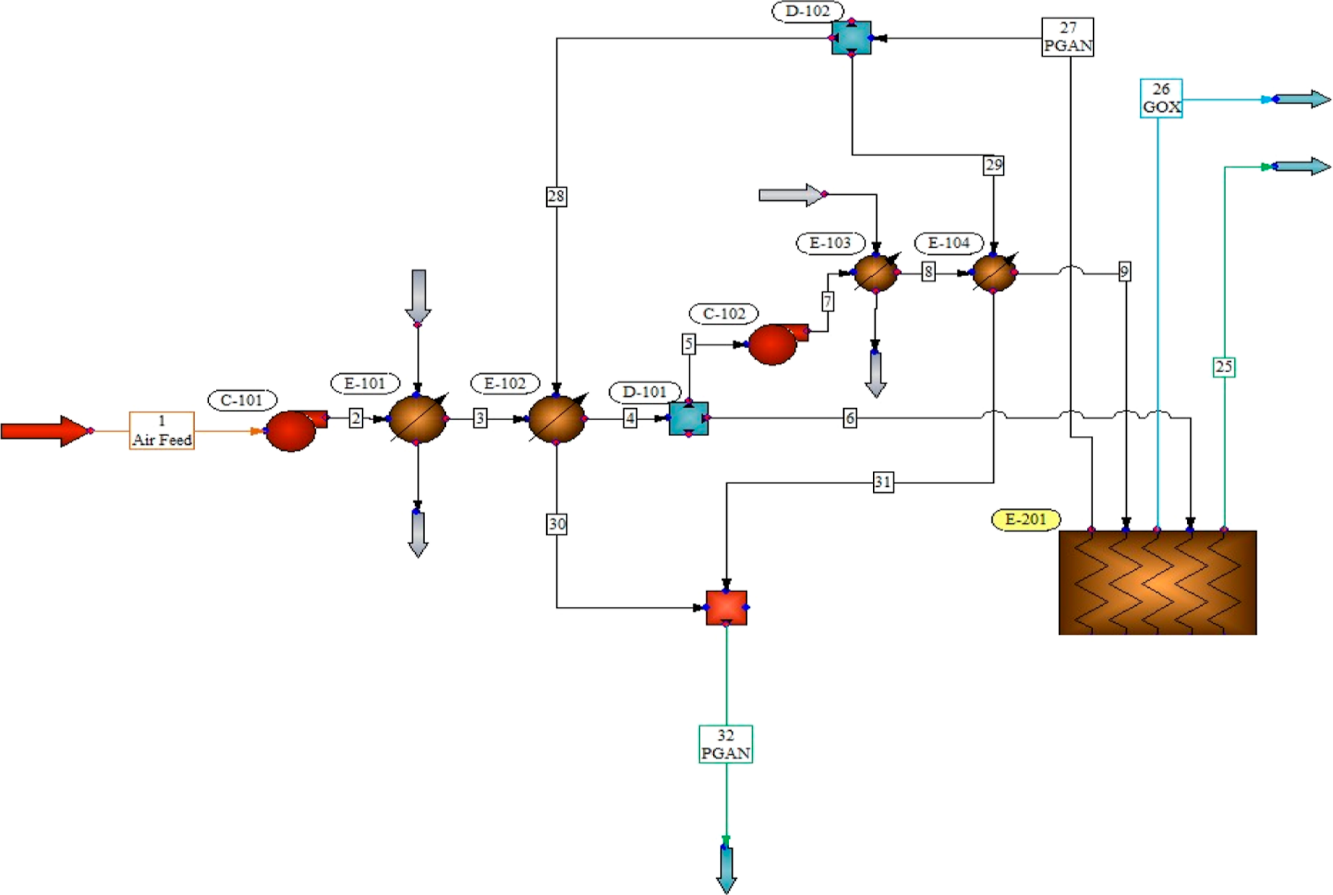
Process flow diagram of the pretreatment of air section.

The study successfully achieved impressive purity levels for the produced substances ranging from 99.49 to 99.99%. These values were obtained directly from the simulation program, indicating the accuracy and reliability of the simulation. The study’s ability to consistently produce high-purity products was a testament to the reliability of the CHEMCAD simulation. This level of precision was crucial for industries where purity standards were stringent, such as in the production of industrial gases. In addition, the study provided detailed information on the flow rates of the produced substances ranging from 5656.02 to 25524.49 kg/h. These quantitative results offered a comprehensive understanding of the production capacity of the cryogenic separation process under the simulated conditions. These specific flow rates served as valuable benchmarks for comparison to future studies or practical applications. It provided practical insights into the potential production scale and efficiency of the cryogenic distillation process. The decision to ignore the warning message was justified by the fact that it did not create any extra negativity about the general operation of the process. This suggested that the warning was related to a specific aspect that did not significantly affect the overall efficiency and performance of the cryogenic separation process. While addressing warnings was crucial, the study’s discernment in determining the impact on the overall process highlights the importance of distinguishing critical issues from those that could have negligible effects on the study objectives. The study also emphasized the reliability of the simulation by confirming that the original values were used in the process. The process flow diagram for the pretreatment of air section in the cryogenic separation process was outlined in the key steps involved in preparing the incoming air stream before it entered the main separation units. Beginning with the intake of ambient air, the process involved initial filtration to remove particulate matter and impurities. The air then underwent compression to elevate its pressure, followed by cooling to lower temperatures. This cooling step was notable for initiating condensation of moisture and certain components. Subsequently, the cooled and compressed air stream was directed through additional purification stages, such as adsorption or absorption processes, to selectively remove specific contaminants. The pretreated air was finally ready for entry into the main cryogenic separation units ensuring that it met the required purity and temperature specifications for optimal separation efficiency. The process flow diagram provided a visual representation of the systematic and controlled steps involved in the initial treatment of the incoming air feed setting the stage for the subsequent cryogenic separation processes (Figure 2).

The initial temperature of 25 °C suggested standard operating conditions for the cryogenic separation process. A pressure of 1 bar and a vapor fraction of 1 indicated a single-phase system. The composition with nitrogen being the predominant component followed by oxygen and a smaller fraction of argon represented typical air composition. These initial conditions provided a baseline for the cryogenic separation process, setting the stage for the subsequent simulation and analysis. The mole fractions of components in the feed stream indicated the relative abundances of nitrogen, oxygen, and argon. Nitrogen constituted 20.95%, oxygen 78.10%, and argon 0.94% of the initial composition. These component mole fractions were crucial for understanding the feed composition and tailoring the cryogenic separation process to achieve the desired purity levels in the product streams. The total flow rate provided a quantitative measure of the feed entering the cryogenic separation unit. This information was essential for determining the process capacity and evaluating the efficiency of the separation process. Understanding the total flow rate was crucial for scaling the process, assessing equipment capacity, and ensuring the feasibility of the separation under given conditions. The relatively high fraction of oxygen in the feed stream indicated the potential for selective separation to achieve high-purity nitrogen and argon products. The lower fraction of argon suggested that it could be a minor component in the product streams. The composition data guided the simulation toward achieving the desired purity levels for nitrogen, oxygen, and argon in the final product streams. The initial temperature of 25 °C and pressure of 1 bar aligned with typical conditions for cryogenic separation processes. These parameters impacted the phase behavior of the components and were critical for determining the process efficiency. The specified temperature and pressure influenced the thermodynamics of the separation process, affecting the critical temperatures and pressures for the components. The vapor fraction of 1 indicated that the initial state was fully vaporized. Understanding the vapor phase was notable for the design and efficiency of the cryogenic separation processes. A vapor phase showed that the cryogenic separation process could involve cooling to temperatures below the critical temperature of the components, allowing for effective separation through condensation. C-101 was the main compressor which compressed the process air at 1 to 5.7 bar with 0.97 efficiency. Temperature of the compressed air increased to 221.33 °C. In the industrial applications, the temperature decreased at the output of the compressor to about 80 °C and then cooled in an exchanger to 15–20 °C.^13^ However, in the simulation program, there was no possible compressor equipment for cooling the compressed air at the outlet. Thus, output temperature stream of the compressor was kept at a calculated temperature of 221.33 °C (Table 2). The provided data indicated the use of two centrifugal compressors, denoted as C-101 and C-102. Both were of the centrifugal type, suggesting a commonality in the compression technology employed. The choice of centrifugal compressors typically implied that efficient compression of large volumes of gas was often favored for applications with varying flow rates. The different pressure ratios between the compressors suggested distinct compression stages, with C-102 likely serving a higher-pressure stage in the process. The substantial temperature increases indicated the compressors’ adiabatic nature and the associated heat generated during compression. Efficient heat dissipation mechanisms could be required to maintain an optimal compressor performance. Both compressors shared a high efficiency of 0.97, indicating effective energy conversion during compression. This suggested that a significant portion of the input power was utilized for actual compression work. The high efficiency values were desirable, signifying minimized energy losses and enhanced overall compressor performance. Compressor C-101 required an actual power of 3570.90 kW while C-102 consumed a lower power of 1411.03 kW. The theoretical power values were 3463.77 and 1368.70 kW, respectively. The actual power exceeding the theoretical power for both compressors indicated some losses likely due to friction and inefficiencies. Monitoring and optimizing power consumption are crucial for energy-efficient operations. The Cp/Cv values for both compressors (1.401 for C-101 and 1.410 for C-102) represent the specific heat ratio indicative of the gas properties during compression. Understanding Cp/Cv was essential for accurately modeling the thermodynamics of the compression process and predicting temperature changes. The calculated adiabatic head for C-101 was 19682.70 m, while for C-102, it was 22186.92 m. Adiabatic head represented the work done during compression under adiabatic conditions. The difference in adiabatic head values reflected the varying compression requirements of the two compressors, with C-102 likely managing higher compression duties.

**Table 2:** Operation Conditions of C-101 and C-102

compressor type	centrifugal (C-101)	centrifugal (C-102)
inlet pressure (bar)	1	5.7
outlet pressure (bar)	5.7	40
inlet temperature (°C)	25	17
outlet temperature (°C)	221.33	238.83
efficiency η_eff_	0.97	0.97
actual power (kW)	3570.90	1411.03
theoretical power (kW)	3463.77	1368.70
*C*_p_/*C*_v_	1.401	1.410
calculated head (adiabatic) (m)	19682.70	22186.92

After the compression of process air, it was first desired to cool the compressed air with a single exchanger which used nitrogen gas produced from the high-pressure column. However, this choice caused an error in the system. Thus, a new option was delivered that suggested the application of a double heat exchanger connected in series to cool the compressed air. First, heat exchanger used cooling water as a cooling agent, where the second exchanger used chilled nitrogen gas produced from the high-pressure column. Majority of the heat exchange occurred in the first exchanger. E-101 was the heat exchanger that was used for cooling the compressed air to a temperature of 30 °C from 221.33 °C. Cooling water at 10 °C and 0.5 bar were used as the cooling agent for this exchanger. A flow of 4509.18 kg/h was calculated from the simulation program for cooling water. Cooling water left the exchanger at 180 °C with a vapor phase. Heat duty for this equipment was calculated from the program as 12,611 MJ/h which was approximately 3503 kW (3.5 MW).

The data indicated the use of four shell-and-tube heat exchangers, E-101, E-102, E-103, and E-104. Shell-and-tube exchangers are common in cryogenic processes for their efficiency in handling high temperature differentials. The choice of this type of exchanger suggested its suitability for the cryogenic separation process, particularly in managing heat exchange between different process streams. The inlet and outlet pressures for both hot and cold streams varied among the four exchangers. Inlet pressures ranged from 0.5 to 40 bar while outlet pressures spanned from 5.3 to 40 bar. These pressure variations indicated that each heat exchanger was tailored to handle specific pressure conditions aligned with the diverse requirements of the cryogenic separation process. The wide temperature ranges reflected the diverse temperature profiles of the hot streams, showcasing the flexibility of these heat exchangers in handling varied process conditions. Temperature differentials signified the heat transfer occurring in the cold streams and the associated cooling or condensation processes facilitated by the heat exchangers. The varying heat duties reflect the diverse roles of each heat exchanger in the cryogenic separation process. E-101, with the highest duty, likely handled a substantial portion of the heat exchange. Operation conditions of heat exchangers are given in Table 3.

**Table 3:** Operation Conditions of Heat Exchangers

exchanger type	shell and tube (E-101)	shell and tube (E-102)	shell and tube (E-103)	shell and tube (E-104)
inlet and outlet pressures of hot stream (bar)	5.7	5.7	40	40
inlet and outlet pressures of cold stream (bar)	0.5	5.3	0.5	5.3
Th_in_ and Th_out_ (°C)	221.33–30	30–17	238.83–70	70–50
Tc_in_ and Tc_out_ (°C)	10–180	–20–19.7	10–150	–20–69.68
calculated heat duty (kW)	3503	236.66	1114.2	133.55

E-102 was connected to the output stream of E-101 to further cool the compressed air to 17 °C which was required to maintain operational conditions.^12^ E-102 differed from E-101 in the usage of chilled nitrogen gas rather than cooling water. Nitrogen gas was produced from the high-pressure column and serviced back to the main heat exchanger (E-201) and exited as pressurized gaseous nitrogen (PGAN). Usage of a back stream for the cooling agent reduced the cost of utilities in the process. Compressed air at 30 °C entered the exchanger as a hot stream while PGAN at −20 °C entered as a cold stream. Both streams were at the vapor phase. The temperature of compressed air decreased to 17.0 °C with a calculated heat duty of 852.79 MJ/h which was approximately 236.66 kW. The temperature of the cold stream increased to 19.7 °C. PGAN was further transferred to a mixer, where two PGAN streams were mixed and then transported to storage tanks. D-101 was a simple device that divided stream no. 4 into two streams with a ratio of 0.35 to 0.65 as stream no. 5 and stream no. 6. Second compressor in the process was used to compress the upcoming stream no. 5 from divider which contained 0.35 of the process air at 5.7 to 40 bar. The elevated pressure could prove beneficial in subsequent stages of the process, particularly when this stream passes through a turbine. An expansion could lower its pressure levels while also lowering its temperature. Thus, in the main heat exchanger (E-201), there could be a lower energy consumption to cool this stream. Compressing the air from 5.7 to 40 bar resulted in its temperature to increase from 17 to 238.83 °C. This level of temperature was not acceptable to enter the cold box. This problem was solved with the addition of two heat exchangers connected in series.

Purpose of using after coolers for the second time was the same as the first one. It was desired to lower the temperature of process air that increased after the compression. Similar equipment was used with the same cooling agents. At the beginning, the temperature of 238.83 °C gradually decreased to 50 °C. E-103 was the first heat exchanger with a utility stream of water cooling after cooling. Likewise in the first system, the majority of the heat exchange occurred in the first exchanger. An inlet stream at 238.83 °C was decreased to 70 °C with an inlet cold stream of cooling water at 10 °C and 0.5 bar. The cold stream left the system at 150 °C. A flow of 1464.83 kg/h of cooling water was needed to maintain the system. Estimation of the flow rate for the cooling water was performed by the simulation program. Also, heat duty was calculated as 4011.12 MJ/h, which was approximately 1114.2 kW (1.11 MW).

**Figure 3:**
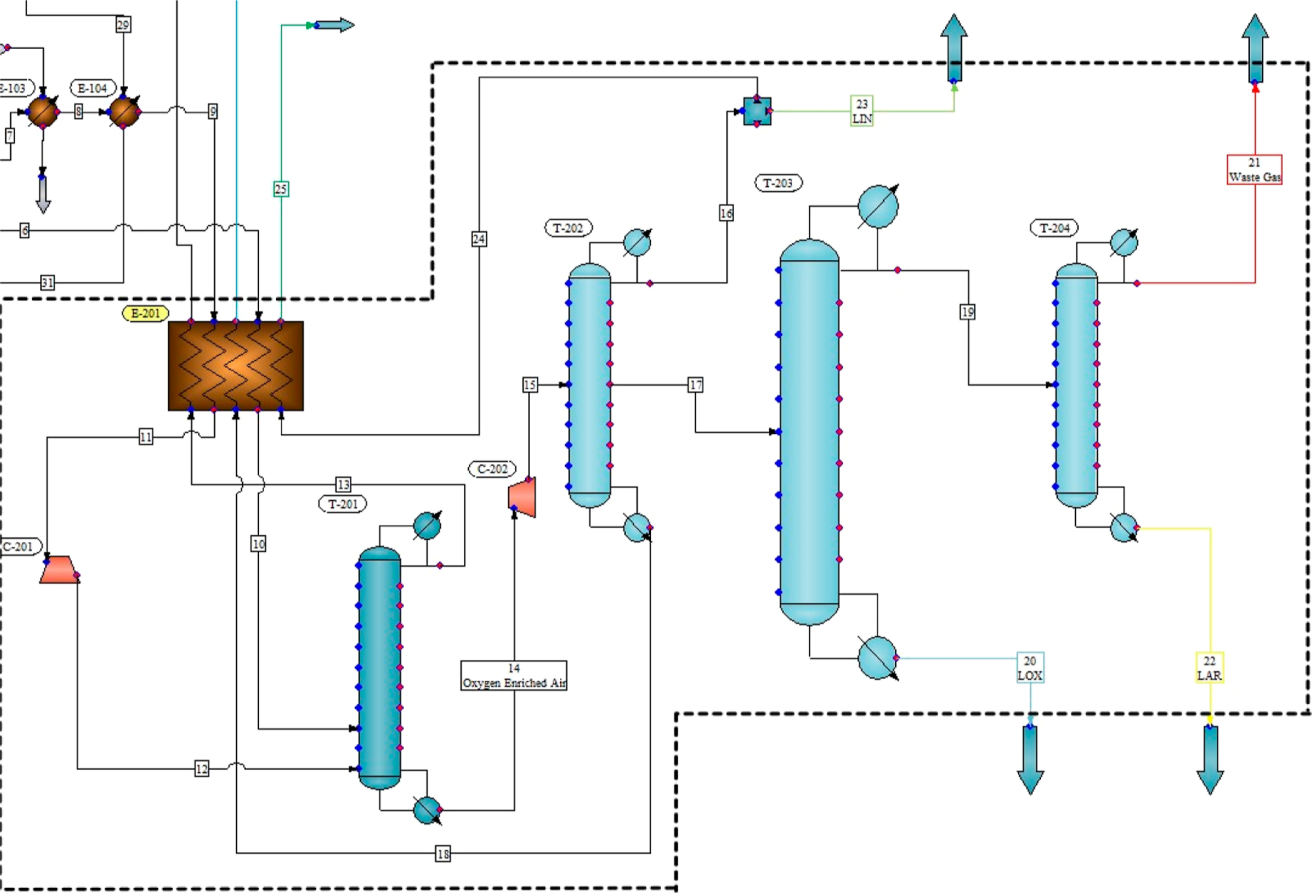
Layout of cold box in the simulation.

The layout of the cold box in the simulation served as a critical component in the cryogenic separation process, where it played a central role in housing the intricate network of heat exchangers, distillation towers, and associated equipment. Typically designed with a focus on spatial efficiency, the cold-box arrangement ensured a systematic flow of cryogenic fluids through the various units. Heat exchangers such as shell-and-tube configurations were strategically positioned to facilitate efficient heat transfer while distillation towers were organized in a manner that optimized separation efficiency. The layout also accommodated auxiliary equipment, such as compressors and pumps, to maintain the required pressures and flows. Overall, the cold box layout was carefully engineered to maximize process performance, minimize energy consumption, and ensure seamless integration of the individual components within the cryogenic separation system (Figure 3).

E-104 further cooled the high-pressure process air from 70 to 50 °C. PGAN produced from the high-pressure column was used as a cooling agent at −20 °C. PGAN left the system at 69.68 °C and then mixed with the cooler utility output of PGAN in a mixer. The flow rate of the PGAN used in the E-104 was 0.2 of the total back serviced stream of PGAN. Heat duty for the equipment was calculated as 480.81 MJ/h which was approximately 133.55 kW (0.13 MW). These two devices were related with the transportation of PGAN. Dividing the back serviced PGAN stream with a division ratio of 0.8 to 0.2 into two streams, streams no. 28 and 29, occurred in D-102 equipment. These two streams were used as cooling agents in E-102 and E-104 to lower the cost of utilities. Temperature of both streams had increased after the heat exchanges. Then, two streams were mixed in M-101 for further transportation to storage tanks as PGAN. The final temperature of the mixed streams was calculated as 29.69 °C by the simulation program at the M-101 outlet. Second part of the process was called a cold box where the cryogenic operations occurred. Two expanders (C-201 and C-202), one multistream exchanger (E-201) and four different sized with different operating pressure columns (T-201, T-202, T-203, and T-204) were used. Separation of air into its components was performed in the towers. Each tower had its own name, which defined their duty in this process as T-201: high-pressure (HP) column, T-202: low-pressure (LP) column, T-203: crude argon (CAR) column, and T-204: pure argon (PAR) column. Main heat exchanger also called multistream exchanger (E-201) handled the heat exchange between the feed streams with the back serviced streams. There were two separate feed streams, which were mentioned earlier that a 0.35 portion of the total feed entered the E-201 at 40 bar and partially cooled where the 0.65 portion of the total feed entered at 5.7 bar and chilled to −178 °C which was nearly the liquefaction point of air. After expansion of the high-pressure air, these two streams entered T-201 from different stages. Tower operated at 5.3 bar, thus called the high-pressure column. Pure nitrogen (99.99%) collected from the condenser where oxygen-enriched liquid air (OELA) (35% O_2_) collected from bottom. Top product was called PGAN. PGAN was then serviced back to E-201 and D-101 to perform heat exchange operations. On the other hand, OELA expanded with C-202 and transferred to the T-202 to further separate the O_2_ from the remaining compounds. Liquid nitrogen (LIN) was collected from the top, and liquid oxygen (LOX 99.8%) was collected from the bottom of the LP column. A gas stream containing nearly about 10% of Ar gas left the column at the intermediate stage. This gas stream was then transferred to a CAR column. This column was the tallest among the other four columns. In the CAR column, the gas containing 10% of Ar and 89.99% O_2_ were separated from each other. Almost all the O_2_ was left at the bottom as liquid while the Ar with significantly low content of N_2_ was left from the top of the column. Further purification of Ar was done in the PAR column where it was possible to collect 99.99% pure Ar product. Top product of the PAR column left the system as a waste stream. There were total of 10 streams flowing into the main heat exchanger also called as the multistream heat exchanger. Two of the inlet streams were hot inlets that supplied from the warm section. Both streams contained process air stream no, 6 and 9 entering the E-201 at 17 and 30 °C, respectively. While the outlet stream of stream 6 (stream 10) left the exchanger at −178 °C, outlet of stream 9 (stream 11) partially cooled to −135.8 °C, heat duty was supplied from the cold streams serviced back from T-201 and T-202. Stream 13 (at −178.2), 18 (at −180.06), and 24 (at −192.72) entered the E-201 from the cold end to perform the duty. All three of these streams were in the liquid phase. After the heat exchange operations, cold end streams left the system at −20 °C (stream 27), 45 °C (stream 26), and 45 °C (stream 25). All the temperature specifications were determined but one was left unspecified to run the simulation. Temperature of stream 11 was obtained by the program as −135.8 °C. Simulation program gave the results of heat duties. Optimum specifications of the heat exchanger are given in Table 4. The variation in temperatures indicated the diverse thermal requirements within the cryogenic separation process. Specified outlet temperatures suggested predefined conditions while calculated values could result from specific heat exchange calculations. The negative sign of the heat duties indicated heat absorption in the process reflecting the cooling or condensation nature of these streams. The varying magnitudes of heat duties showed different thermal loads on the heat exchangers. The heat duty per unit temperature change (specific heat absorption) varies for each stream based on the provided data. Understanding the specific heat absorption was significant for evaluating the efficiency of the heat exchange process. Higher values indicated greater heat absorption per degree change in temperature. The specified outlet temperatures for some streams suggested predefined process requirements. In contrast, the calculated outlet temperature for stream 9 indicated a dynamically determined condition based on the heat exchange process. The mix of specified and calculated outlet temperatures emphasized the dynamic and controlled nature of the cryogenic separation process where some conditions were predetermined. Net heat duty was calculated as 0.04 MJ/h. This formed a situation that an additional energy input was required for the system. Although the simulation worked without any error, a single warning had been encountered in this equipment.

**Table 4:** Specifications of E-201

inlet stream	inlet T(°C)	outlet stream	outlet T (°C)	heat duty (MJ/h)
**6**	17	**10**	–178 (specified)	–16064.8
**9**	30	**11**	–135.8 (calculated)	–4968.29
**13**	–178.24	**27**	–20 (specified)	8831.94
**18**	–180.06	**26**	45 (specified)	3882.43
**24**	–192.77	**25**	45 (specified)	8318.68

Specified values for each stream were changed to overcome this warning but it could not succeed. For the simulation of all distillation columns, it was first designed with the shortcut distillation equipment in the program. Design parameters of pressure of the column, split of low-key (LK) and heavy key (HK) components, and *R*/*R*_min_ value were specified. After the specifications, the program simulated and calculated the required number of stages, feed location, and reflux ratio to perform separation with the desired split of components. Obtained parameters were then used in the trayed tower equipment with reboiler and condenser to perform a more precise approach to the separation process. The separation process began with the T-101 column. The column is also called a high-pressure column because it operated at 5.3 bar. Two separate streams were entered into the distillation column. Stream 10 entered stage 50 and stream 12 entered stage 58. Total of 60 stages were calculated by the program. N_2_ of 99.99% purity (stream 13) was collected from condenser at −178.24 °C and then serviced back to exchange heat with the process air in the E-201. From bottom (stream 14), OELA was collected at −174.56 °C. It was called OELA because it contained O_2_ about 35%. Stream 14 was then transferred to the LP pressure column to further separate oxygen from the other components (Table 5). The simulation involved four distillation towers, all the trayed type. Each tower had a different number of stages. The varying number of stages indicated different separation requirements and complexities for each tower tailored to specific components and purity goals. The choice of feed stages was significant for introducing the feed streams into the towers at optimal locations facilitating efficient separation. Varied column pressures indicated different pressure conditions for achieving the desired separation and vapor–liquid equilibrium in each tower. The reflux ratio was a key parameter in distillation influencing separation efficiency. Higher reflux ratios generally lead to better separation but may require more energy. The temperature profiles presented insights into the thermal conditions within the towers, influencing the separation of components. Negative values suggested heat absorption in the condenser contributing to the cooling and condensation of vapor. The magnitude indicated the intensity of heat exchange. Positive values defined heat input to the reboiler for vaporization, essential for maintaining the distillation process by generating vapor for separation.

**Table 5:** Unit Operation Table for Inlet and Outlet Streams of Tower from the Simulation Program

tower type	trayed	tower type	trayed	tower type	trayed	tower type	trayed
number of stages	60	number of stages	30	number of stages	191	number of stages	22
1st feed stage	50	feed stage	12	feed stage	141	feed stage	7
2nd feed stage	58	draw stage for gas stream	19	column ressure (bar)	1.3	column pressure (bar)	1.3
column pressure (bar)	5.3	column pressure (bar)	1.4	calculated reflux ratio	35	calculated reflux ratio	6
calculated reflux ratio	1.2	calculated reflux ratio	11.6	estimated top temperature (°C)	–183.47	estimated top temperature (°C)	–193.42
estimated top temperature (°C)	–176.17	estimated top temperature (°C)	–192.59	estimated bottom temperature (°C)	–180.84	estimated bottom temperature (°C)	–183.45
estimated bottom temperature (°C)	–174.54	estimated bottom temperature (°C)	–180.07	calculated condenser duty (MJ/h)	–4266.95	calculated condenser duty (MJ/h)	–50.216
calculate condenser duty (MJ/h)	–9908.28	calculated condenser duty (MJ/h)	–57686.42			calculated reboiler duty (MJ/h)	50.210
calculated reboiler duty (MJ/h)	6190.27	calculated reboiler duty (MJ/h)	57946.25				

**Figure 4:**
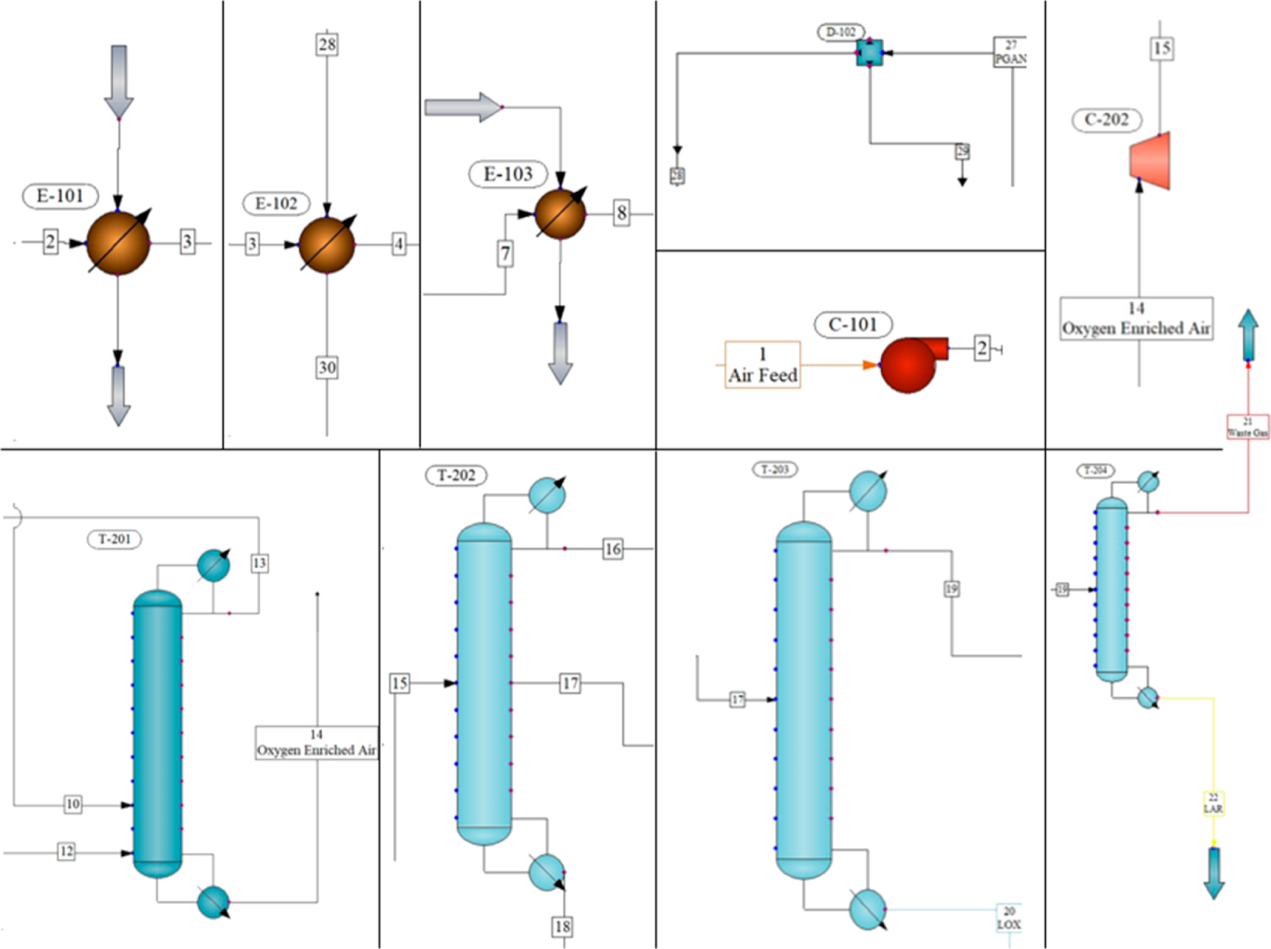
CHEMCAD images utilized in the process.

In the second distillation column, OELA (stream 15) was separated to further purify O_2_. OELA entered the distillation column at −174.56 °C and 1.4 bar. It was separated into three different streams (streams 16, 17, and 18). Same procedure with the first column was used for the estimation of the number of stages. As a result, the program calculated the required number of stages as 30 and location of feed at stage 12, respectively. Top product (stream 16) contained 99.90% pure N_2_ at −192.77 °C. This stream was divided into two streams (streams 23 and 24). Stream 23 contained 20% of the total LIN and was directly transferred to storage tanks where stream 24 was serviced back to exchange heat with the incoming hot process air in the E-201. At the intermediate stage, a gas stream (stream 17) was collected that contained nearly 10% of Ar. The location of the draw stage was determined after several attempts. Simulation was not able to calculate the draw stage. It was aimed to get maximum level of Ar content, and it was achieved at stage 19 of the column. Stream 17 left the system at −180.38 °C and then transferred to the crude argon (CAR) to eliminate the O_2_ content of 89.9%. The bottom stream (stream 18) was collected from the reboiler at −180.07 °C and contained 99.8% of O_2_ in the liquid phase. This stream was also serviced back to exchange heat with the hot process air in the E-201. Gas stream (stream 17) from the outlet of T-202’s intermediate stage entered the T-203 to eliminate O_2_. This column was more sensitive than the other three columns because the boiling points of Ar and O_2_ were significantly close to each other (T_bAr_: −185 °C, T_bO2_: −182 °C). Same procedure with the first column was used for the estimation of number of stages. However, the calculated reflux ratio was nearly 350. To achieve a more applicable system to reality, the reflux ratio was determined as 35. Decrease in the reflux ratio increased the number of stages to 191. Location stage of the feed stream was calculated as stage 141. Top product (stream 19) of the T-203 contained 99.91% Ar and 2 ppm of O_2_. Remaining fraction of the stream contained N_2_ which could be eliminated in PAR (T-204). Stream 19 left the system at −183.46 °C (1.3 bar) and transferred to the PAR. Bottom product (stream 20) contained 99.49% of O_2_ at the liquid phase (LOX) and was directly transferred to storage tanks. The final distillation column separated Ar from N_2_. N_2_ fraction of the feed stream (stream 19) to the column was 0.00084, and the purity of Ar was 99.91%. It was desired to achieve 99.99% purity of Ar. It was the reason why a second column was required to produce Ar. Same procedure with the first column was utilized for the estimation of number of stages. As a result, the program calculated the required number of stages as 22 and location of feed at stage 7. Purity of 99.99% for Ar was achieved at the bottom, but the top product also contained Ar with a fraction of 0.98. Although the flow rate of the top product was about 5% of the flow rate of the feed, this result might not be accepted in plant applications. Bottom product left the system at −183.45 °C and was transferred to liquid storage tanks as liquid argon (LAR). CHEMCAD images utilized in the simulation program are shown in Figure 4.

The difference was in the compressors used to compress the air. The process of cooling the air at the compressor outlet was not implemented due to the lack of suitable equipment in the simulation program, but instead, additional heat exchangers were added to the compressor outlets and this cooling process was provided partly with water and partly with nitrogen gases which were provided with backflow. Also, since the double column system was not included in the program, an alternative method had been followed. These columns, which were integrated with each other in industrial applications, were used as two separate columns in the simulation. Another detail that could be noticed was the difference in the amount of argon obtained from the intermediate zone of the low-pressure column. It was said that the gas collected from this section contained around 25% argon, but the maximum argon content achievable in this simulation was around 10%. It could be said here that the differences in the type or properties of the columns used could lead to such confusion.^17^ In summary, data analysis delved into the management of warning messages, the achieved purity levels, substance production flow rates, and the overall impact on the general operation of the cryogenic separation process. These insights provided a more nuanced understanding of the study’s outcomes and contributed to the robustness of the conclusions drawn from the CHEMCAD simulation.

## 5 Conclusions

As a general conclusion, it was seen that simulation of the intended process was successfully completed. No error message was received when the simulation was run. A warning message was received for only one E-201 equipment. This was a warning message that there was a possible pinch zone present in the equipment. This warning was due to the large temperature difference that could occur in the heat exchanger. Since it did not create any extra negativity about the general operation of the process, this warning had been ignored by giving the determined values. It was achieved to produce:

- 99.49% LOX with a flow of 5656.02 kg/h
- 99.9% LIN with a flow of 4664.66 kg/h
- 99.99% PGAN with a flow of 25524.49 kg/h
- 99.99% LAR with a flow of 699.94 kg/h

In the simulated cryogenic separation process, the distillation towers emerged as pivotal components, each tailored to specific separation objectives. Notably, the diverse number of stages in the towers ranging from 22 to 191 reflected the nuanced requirements for achieving optimal separation and purity levels for the different components. The selection of feed stages strategically defined feed streams into the towers, contributing to efficient separation. The calculated reflux ratios spanning from 1.2 to 35 underscored the careful balance between the separation efficiency and energy consumption. Temperature profiles revealed that the thermal dynamics within the towers influenced the phase transitions critical for component separation. The calculated condenser and reboiler duties signified the intense heat exchange occurring in the system, with varying magnitudes indicative of the specific energy demands for each tower. Collectively, these results highlighted the intricacies of the distillation process, showcasing its adaptability to diverse components and emphasizing the critical role of each tower in achieving the overall objectives of the cryogenic separation unit.

In conclusion, the study’s in-depth exploration of the cryogenic distillation process using CHEMCAD simulation, combined with the integration of the technical literature, marked a significant advancement in the field. Future research could focus on scalability, economic feasibility, environmental impact, and further optimization of the cryogenic distillation process, paving the way for practical applications and industry innovations.

## Data Availability

The data sets generated during and/or analyzed during the study are available from the corresponding author on reasonable request.

## References

[ref1] Shahzad M. W.; Burhan M.; Ang L.; Ng K. C. Energy-water-environment nexus underpinning future desalination sustainability. Desalination 2017, 413, 52–64. 10.1016/j.desal.2017.03.009.

[ref2] Schach M.; Oyarzún B.; Schramm H.; Schneider R.; Repke J. Feasibility study of CO_2_ capture by anti-sublimation. Energy Procedia 2011, 4, 1403–1410. 10.1016/j.egypro.2011.02.005.

[ref3] Zanganeh K. E.; Shafeen A.; Salvador C. H. CO_2_ capture and development of an advanced pilot-scale cryogenic separation and compression unit. Energy Procedia 2009, 1 (1), 247–252. 10.1016/j.egypro.2009.01.035.

[ref4] Yerolla R.; Muhammed R. C. A.; Naseef Y.; Besta C. S. Simulation of cryogenic distillation of atmospheric air using aspen hysys. IFAC-PapersOnLine 2022, 55 (1), 860–865. 10.1016/j.ifacol.2022.04.141.

[ref5] Prashanth K.; Shaik A.; Srinivasa Rao T.; Pavan Bharadwaja B. Experimental investigation of argon gas induction on diesel engine performance and emission characteristics: A comprehensive study on de-NO_x_ techniques. Process Saf. Environ. Prot. 2021, 152, 471–481. 10.1016/j.psep.2021.06.036.

[ref6] Knapik E.; Kosowski P.; Stopa J. Cryogenic liquefaction and separation of CO_2_ using nitrogen removal unit cold energy. Chem. Eng. Res. Des. 2018, 131, 66–79. 10.1016/j.cherd.2017.12.027.

[ref7] Kancherla R.; Nazia S.; Kalyani S.; Sridhar S. Modeling and simulation for design and analysis of membrane-based separation processes. Comput. Chem. Eng. 2021, 148, 107258 10.1016/j.compchemeng.2021.107258.

[ref8] Luca A.; Petrescu L. Membrane technology applied to steel production: Investigation based on process modelling and environmental tools. J. Clean. Prod. 2021, 294, 126256 10.1016/j.jclepro.2021.126256.

[ref9] Ma Y.; Cui P.; Wang Y.; Zhu Z.; Wang Y.; Gao J. A review of extractive distillation from an azeotropic phenomenon for dynamic control. Chin. J. Chem. Eng. 2019, 27 (7), 1510–1522. 10.1016/j.cjche.2018.08.015.

[ref10] Wodołażski A.; Smoliński A. Modelling and process integration study of dimethyl ether synthesis from syngas derived from biomass gasification: Flowsheet simulation. Alex. Eng. J. 2020, 59 (6), 4441–4448. 10.1016/j.aej.2020.07.050.

[ref11] Coker A. K. Distillation; Elsevier eBooks, 2010; pp 1–268.

[ref12] Dimian A. C.; Bildea C. S.; Kiss A. A. Synthesis of separation systems. Comput.-Aided Chem. Eng. 2014, 35, 345–395. 10.1016/B978-0-444-62700-1.00009-7.

[ref13] Sánchez A.; Castellano E.; Martín M.; Vega P. Evaluating ammonia as green fuel for power generation: A thermo-chemical perspective. Appl. Energy 2021, 293, 116956 10.1016/j.apenergy.2021.116956.

[ref14] Aljaghoub H.; Alasad S.; Alashkar A.; AlMallahi M. N.; Hasan R.; Obaideen K.; Alami A. H. Comparative analysis of various oxygen production techniques using multi-criteria decision-making methods. Int. J. Thermofluids 2023, 17, 100261 10.1016/j.ijft.2022.100261.

[ref15] Smith A. M.; Klosek J. A review of air separation technologies and their integration with energy conversion processes. Fuel Process. Technol. 2001, 70 (2), 115–134. 10.1016/S0378-3820(01)00131-X.

[ref16] Gutiérrez F. A.; García-Cuevas L. M.; Sanz W. Comparison of cryogenic and membrane oxygen production implemented in the Graz cycle. Energy Convers. Manage. 2022, 271, 116325 10.1016/j.enconman.2022.116325.

[ref17] Häring H. W. Industrial Gases Processing; John Wiley & Sons: Germany, Darmstadt, 2008.

[ref18] Deng M.; Zhang Q.; Huang Y.; Zhang X. Integration and optimization for a PEMFC and PSA oxygen production combined system. Energy Convers. Manage. 2021, 236, 114062 10.1016/j.enconman.2021.114062.

[ref19] Capstick S.; Bulfin B.; Naik J. M.; Gigantino M.; Steinfeld A. Oxygen separation via chemical looping of the perovskite oxide Sr_0.8_Ca_0.2_FeO_3_ in packed bed reactors for the production of nitrogen from air. Chem. Eng. J. 2023, 452, 139289 10.1016/j.cej.2022.139289.

[ref20] Chemcad Version 8.0 by Chemstations, Inc. 2023, https://www.chemstations.com/.

